# Studying learning in the healthcare setting: the potential of quantitative diary methods

**DOI:** 10.1007/s40037-015-0199-3

**Published:** 2015-07-17

**Authors:** Yvette Ciere, Debbie Jaarsma, Annemieke Visser, Robbert Sanderman, Evelien Snippe, Joke Fleer

**Affiliations:** 1 Department of Health Sciences, University Medical Center Groningen, University of Groningen, FA12, PO Box 196, 9700 AD Groningen, The Netherlands; 2 Center for Education Development and Research in Health Professions, University Medical Center Groningen, University of Groningen, Groningen, The Netherlands; 3 Department of Psychology, Health and Technology, University of Twente, Enschede, The Netherlands; 4 Interdisciplinary Center Psychopathology and Emotion regulation, University of Groningen, University Medical Center Groningen, Groningen, The Netherlands; 5 School for Mental Health and Neuroscience, Maastricht University, Maastricht, The Netherlands

**Keywords:** Quantitative research methods, Diary method, Real-time measurement, Longitudinal

## Abstract

Quantitative diary methods are longitudinal approaches that involve the repeated measurement of aspects of peoples’ experience of daily life. In this article, we outline the main characteristics and applications of quantitative diary methods and discuss how their use may further research in the field of medical education. Quantitative diary methods offer several methodological advantages, such as measuring aspects of learning with great detail, accuracy and authenticity. Moreover, they enable researchers to study *how* and *under which conditions* learning in the health care setting occurs and *in which way* learning can be promoted. Hence, quantitative diary methods may contribute to theory development and the optimization of teaching methods in medical education.

## Introduction

Over the past decade, research in medical education has shifted its focus from outcomes of teaching methods to the processes that constitute learning [1]. This line of research involves questions such as ‘how do people learn?’, ‘what is the influence of contextual factors on learning?’, and ‘how do people respond to different teaching methods’? Researchers have used a wide range of methods to study such questions, including surveys, observational methods (e.g., video recordings) and qualitative diaries (e.g., audio diaries, reflective diaries) [1, 2]. Although each of these methods has its own advantages, a common disadvantage is that they give limited insight into how learning is expressed in daily life, and how aspects of learning interact and unfold over time. Yet, more insight into these daily processes may contribute to a better understanding of the nature, antecedents and correlates of learning in the health care setting.

A method that is not yet widely used in the field of medical education but that may greatly benefit the study of learning is the quantitative diary method. Also referred to as ‘intensive longitudinal methods’ [3] or ‘ecological momentary assessment’ [4], quantitative diary methods involve the repeated measurement of aspects of peoples’ experience of daily life [4]. Subjects are asked to report on their environment (e.g., presence of others), perceptions of their environment (e.g., stressfulness of a situation) and internal states (e.g., mood, motivation) while they are living everyday life. In contrast to other longitudinal methods, quantitative diary methods follow an intensive measurement schedule with measurements taking place daily or even multiple times a day during a period of several days or weeks [3, 4]. Hence, they may offer detailed insight into when and how learning phenomena are experienced, how they vary over time and across different situations, and into the factors that trigger, accompany or follow them [5].

The goal of this article is to give an introduction to quantitative diary methods and explain how their use may contribute to the study of learning in the health care setting. First, the key characteristics of quantitative diary methods are described. Second, different types of diary study designs are reviewed. Third, the type of research questions that can be addressed with a diary study are discussed as well as the main advantages and disadvantages of quantitative diary methods. This article does not aim to give a comprehensive overview of diary methodology nor to provide an expert opinion. Instead, the aim of this article is to familiarize the reader with the diary method and to serve as a guide to further reading on this topic.

## Quantitative diary methods

Since quantitative diary studies are used to measure subjective phenomena (i.e., experience), they typically involve self-report [6]. In the educational setting, diaries may be used to measure subjective aspects of learning, such as perceptions of a learning situation. In the case of more objective outcomes (observable behaviour, physiological parameters), diary studies have also incorporated ‘ambulatory assessment’, i.e., the use of monitoring devices such as accelerometers or blood pressure monitors [4]. In medical education, these may be used to measure, for instance, physiological stress responses to learning situations.

Most often, diaries contain a number of pre-defined questions to which subjects respond on a numerical rating scale (e.g., a Likert scale) [7]. Sometimes, numerical questions are combined with open questions to elicit information (e.g., location, activity) that is later coded by the researcher to obtain categories [8]. In earlier diary studies, diary questions were mostly presented in paper-and-pencil booklets. However, since the introduction of palmtop computers, PDAs, and (more recently) smartphones, researchers have increasingly made use of electronic diaries [4, 8, 9].

The number and timing of measurement points varies greatly between studies. The choice of a measurement schedule is generally based on the phenomenon under study as well as the burden for participants [6]. For example, in ‘daily diary studies’, subjects complete a diary at the end of the day [4]. While such a design presents a relatively low burden to participants, it is less suitable for studying phenomena that fluctuate throughout the day and are likely to be inaccurately recalled (e.g., mood, stress perception) [6]. In the case of such phenomena, one may consider a design with multiple measurements per day. For example, in studies using ‘the Experience Sampling Method’ [8, 10], subjects report on their current (‘momentary’) experience at multiple (e.g., 10) moments per day [8]. Hence, these studies do not require recall and give information about fluctuations in phenomena during the day. However, the measurement schedule may be more burdensome for participants.

## Types of quantitative diary study designs

Two types of quantitative diary designs can be distinguished: event-based and time-based designs [3, 4]. In *event-based designs*, measurements only take place after a pre-defined situation or event has occurred. This type of design is useful when one is interested in the prevalence of a particular event, or subjects’ responses to this event (e.g., students’ experience of stress when engaging in a certain learning activity) [6]. On the other hand, *time-based designs* are more appropriate when one is interested in ongoing experiences (such as mood) over time, independent of the occurrence of a particular event [6].

Time-based designs can either follow a fixed or a variable measurement schedule [3, 4]. In both cases, they may involve the use of signalling to prompt subjects to complete a measurement [4]. In *fixed* time-based designs, measurements are scheduled at set time points that are known to the participant. Measurements can be scheduled at equally spaced intervals (e.g., every 2 h) or at specific moments of the day (e.g., around mealtimes, before bedtime). In time-based designs with a *random schedule*, measurements take place at random times that are unknown to the participant [8, 11]. Random schedule designs can be used to obtain a representative sample of moments within the subject’s day and reduce bias due to anticipation of measurement [4, 8]. Generally, random schedule designs involve the measurement of momentary experience, such as is the case in the Experience Sampling Method [12].

## Type of research questions

Quantitative diary methods have been used for broadly three purposes: (a) to study the nature of a phenomenon, (b) to examine variation in a phenomenon within individuals over time, and (c) to study the factors that explain variation in a phenomenon [3, 4]. These three different applications are illustrated below using examples of diary studies from both clinical and education research.

### (a) Studying the nature of phenomena.

One application of quantitative diary methods is to obtain an accurate and detailed account of when and how often a phenomenon occurs in daily life [3]. For instance, quantitative diaries could be used to gain insight into the degree to which students engage in certain learning activities (e.g., searching for literature). An example of this application is provided by Murray et al. [13], who used log diaries to examine how much time students in an internal medicine clerkship spent on different learning activities. The study compared activities in a hospital setting and in general practice and found that students in general practice spent less time on unsupervised interaction with patients and more time on assessment [13]. Thus, by using a diary design, this study was able to shed light on what students actually do in a medical clerkship and to compare two different learning settings.

In the example above, time spent on learning activities could also have been examined with a retrospective method (e.g., a questionnaire), but this would have involved students recalling their time spent on these activities at a later point in time. The accuracy of such a retrospective report is likely to be impaired by memory bias [14]. For example, students may base their report on a typically busy day and therefore overestimate their time spent on study activities. Because in diary studies phenomena are measured closer to their actual occurrence, reports are expected to be more accurate (i.e., reliable) [14, 15]. Furthermore, since phenomena are measured across a range of situations in daily life, findings may be more authentic (i.e., ecologically valid) [4, 8, 16].

### (b) Examining variation within individuals over time.

A second purpose of quantitative diary methods is to study variability or change in a phenomenon within individuals over time [3]. This may involve studying the natural course of learning or change in response to an intervention.

Moment-to-moment variability within individuals may give insight into the extent to which learning is influenced by factors such as the learning environment or an individual’s psychological state. For example, Ahmed et al. [17] used a diary design to study day-to-day variation in the mood of secondary school students. During a period of two weeks, students completed paper-and-pencil diaries four times a week after a mathematics class. Results showed that there was substantial variation in emotions within individuals across days. This finding indicates that students may respond differently to different classroom demands, which would not have been revealed with a single measurement [17].

Due to the high density of measurements, quantitative diaries may also capture micro-level changes in learning that are difficult to capture with designs using fewer observations. For example, a pre-post design may not demonstrate reductions in average stress levels after an intervention, whereas a diary study may show that subjects are indeed able to cope better with particular events. An example of this application in clinical research is provided by Barge-Schaapveld and Nicolson [18], who used a diary method to study the effects of antidepressant treatment versus placebo on quality of life. They found that patients receiving antidepressant treatment did not show greater increases in quality of life than those receiving a placebo. However, patients receiving antidepressants did show fewer within-person fluctuations in quality of life [18]. Thus, this study revealed a treatment effect that would not have been detected using a design with fewer measurements (e.g., a pre-post design).

### (c) Explaining variation within and between individuals.

A third application of quantitative diaries is to study factors that explain variation in a phenomenon. First, one may look at the factors that explain why phenomena vary from one moment to another. For example, what are the conditions under which students experience more or less stress during their clerkships? Second, one may look at factors that explain why individuals differ from each other. For example, what characterises the students that experience most stress during their clerkships? Interestingly, quantitative diary studies also permit examination of the interaction between within-subjects and between-subjects variables. For example, do some students respond differently to certain stressful situations (e.g., transition between clerkships) than others? Studying factors that explain variation in phenomena may help to gain a better understanding of how and when people learn best, which may inform the development of theory and interventions.

There are two ways in which one can study factors that explain within-person variation. The first is by examining cross-sectional associations, i.e., associations between variables that co-occur at the same time point [4]. For example, Stucky et al. [19] studied cross-sectional associations to identify factors that explain why physicians experience more work stress on some days than on others. Participants carried handheld devices for up to seven days. They completed questions about sleep and workplace factors at the beginning of workdays. In addition, they answered questions about stress and work activities in response to random beeps throughout the day. Findings showed that physicians experienced more stress when they had a higher number of patients assigned and the quality of their sleep in the previous night was lower. Furthermore, on average, interns reported higher stress levels compared to residents and attending physicians [19]. Thus, by looking at factors that explain variation within individuals, this study highlighted factors that contribute to the experience of work stress and may need to be targeted when trying to reduce work stress in physicians. Furthermore, by assessing between-subject effects, this study identified a subgroup of physicians that is most at risk for experiencing stress (i.e., interns).

The second way to explain within-person variation is by studying temporal associations, i.e., associations between a variable of interest and a variable that precedes the presence of (or change in) this variable [4]. For example, Snippe et al. [20] studied temporal associations to examine whether changes in mindfulness and repetitive thinking preceded, as suggested by theory, changes in depressive symptoms within the context of an 8-week mindfulness-based intervention for depression. Participants completed daily diary questionnaires from the start of treatment until the last session of treatment. Although changes in mindfulness and repetitive thinking were only associated with change in depressive symptoms in a subgroup of participants, the researchers did not find evidence for reverse causality (i.e., that change in depressive symptoms precedes change in mindfulness and repetitive thinking). Thus, by using a diary design, the researchers were able to test a theoretical assumption about how mindfulness-based interventions help to reduce depression [20].

## Advantages and disadvantages

In sum, quantitative diary methods may offer insight into the nature of learning phenomena by allowing researchers to obtain accurate and authentic observations of (aspects of) learning in daily life. Furthermore, by revealing variation within individuals over time, they may give insight into the extent to which learning is influenced by other conditions (e.g., the environment, mental state) and capture micro-level changes in learning that may not be captured using designs with fewer measurements. Finally, quantitative diary methods can be used to uncover variables that precede or accompany learning.

With this introduction of quantitative diary methods we respond to recent calls for more quantitative methods in medical education research that were published in this journal (i.e. McKinley [21], Merrienboer [22], and Goldszmidt [23]. For instance, they are useful for testing theoretical assumptions and the effectiveness of theory-based interventions, as was called for by McKinley [21]. Quantitative diary methods may, however, in particular show promise in overcoming the problems associated with the measurement of subjective aspects of learning, as identified by van Merrienboer [22] and Goldszmidt [23]. First, since experience is measured close to the actual occurrence of events, diary methods involve less recall than retrospective methods such as interviews or questionnaires. Second, whereas in interviews or questionnaires participants are generally asked to reflect on processes, diary methods allow observation of the components of these processes (e.g. emotions, cognitions, behaviours), and their interaction in daily life, without requiring reflection of the participant. Third, in contrast to other naturalistic data collection methods, quantitative diaries enable data collection in the absence of the researcher, which reduces the risk of participants giving socially desirable answers.

Nevertheless, the benefits gained from a quantitative diary method are strongly dependent on the choice of a research design that fits the research question and phenomenon under study [3]. For guidance on the design of diary studies, the reader is referred to text books by Stone and Shiffman [24], Hektner et al. [8] and Bolger and Laurenceau [9].

As with any other method, quantitative diary methods also come with several limitations. The frequency and intrusiveness of measurements may present a burden to participants [3, 5], which may result in self-selection of highly motivated and better functioning participants [3, 25]. In addition, high participation burden may lead to non-compliance with the measurement schedule, missing data, and attrition [4, 5, 25]. Finally, researchers need to be aware of the issue of reactivity; behaviour or experience may be influenced by self-monitoring it [3–5, 25]. For advice regarding measures to minimize the impact of the above-mentioned problems, the reader is referred to publications by Shiffman et al. [4], Palmier-Claus et al. [7], and Scollon et al. [25].

## Conclusion

The purpose of this article was to highlight the potential contribution of quantitative diary methods to medical education research. Quantitative diary methods may benefit the study of learning in the health care setting by allowing researchers (a) to obtain a rich description of when and how often learning phenomena occur, (b) to observe how learning phenomena vary across different situations and change over time, and (c) to study factors that precede and accompany learning outcomes. By enabling researchers to study *how* and *under which conditions* learning occurs and *in which way* teaching methods may promote learning, diary studies may advance both research and practice.

### Conflict of interest

The authors report no conflicts of interest.
